# Reference Expression Profile of Three *FBN1* Transcript Isoforms and Their Association with Clinical Variability in Marfan Syndrome

**DOI:** 10.3390/genes10020128

**Published:** 2019-02-11

**Authors:** Louise Benarroch, Mélodie Aubart, Marie-Sylvie Gross, Pauline Arnaud, Nadine Hanna, Guillaume Jondeau, Catherine Boileau

**Affiliations:** 1 Laboratory for Vascular Translational Science, INSERM U1148, Centre Hospitalo-Universitaire Xavier Bichat, 46 rue Henri Huchard, 75018 Paris, France; louise.benarroch@inserm.fr (L.B.); melodie.aubart@inserm.fr (M.A.); marie-sylvie.gross@inserm.fr (M.-S.G.); pauline.arnaud@inserm.fr (P.A.); guillaume.jondeau@inserm.fr (G.J.); 2 Service de Neuropédiatrie, Hôpital Necker-Enfants-Malades, 149 rue de Sèvres, 75015 Paris, France; 3 Département de Génétique, Centre Hospitalo-Universitaire Xavier Bichat, 46 rue Henri 17 Huchard, 75018 Paris, France; nadine.hanna@aphp.fr; 4 Centre de Référence pour le Syndrome de Marfan et syndromes apparentés, Service de Cardiologie, Centre Hospitalo-Universitaire Xavier Bichat, 46 rue Henri Huchard, 75018 Paris, France; 5 UFR de Médecine, Université Paris Diderot, 16 rue Henri Huchard, 75018 Paris, France

**Keywords:** Marfan syndrome, clinical variability, fibrilline-1, alternative splicing, isoforms

## Abstract

Marfan syndrome (MFS) is a rare connective tissue disorder mainly due to mutations in the *FBN1* gene. Great phenotypic variability is notable for age of onset, the presence and absence, and the number and the severity of the symptoms. Our team showed that *FBN1* gene expression level was a good surrogate endpoint for severity of some MFS clinical features. Eight alternative transcripts are referenced for the *FBN1* gene. We hypothesized that MFS clinical variability could be related to specific *FBN1* isoforms. Isoform expression profiles were investigated in skin and adventitial fibroblasts from controls and MFS patients. The results of the study showed that, in skin and adventitial fibroblasts, only three isoforms were found: *FBN1_001*, *FBN1_004*, and *FBN1_009*. The main isoform was *FBN1_001* and it was significantly reduced in skin and adventitial fibroblasts of MFS patients. The expressions of *FBN1_004* and *FBN1_009* isoforms were similar between controls and MFS patients. However, the expression of the three isoforms was correlated only in patients. Furthermore, their expression levels were associated with the presence of ectopia lentis in MFS patients. Therefore, our results highlight that the two minor alternatively spliced *FBN1* isoforms play a possible role in the pathogenesis of the disease.

## 1. Introduction

Marfan syndrome (MFS; Online Mendelian Inheritance in Man database (OMIM) #154700) is an autosomal dominant connective tissue disorder with an estimated prevalence of one in every 5000 individuals. It is a multisystemic disease that affects the ocular, skeletal, and cardiovascular systems, as well as lung, skin, and dura. In most cases, MFS is due to mutations in the *FBN1* gene encoding fibrillin-1, an extra-cellular matrix protein. To date, over 3000 mutations were reported in the *FBN1* database [1]. The syndrome displays great clinical variability both between and within families, with respect to the age of onset, the presence or the absence, and the number and the severity of the symptoms. This variability is taken into account in the last nosology for MFS with which the diagnosis can be made with or without ectopia lentis or aortic aneurysm [2]. Clinical variability cannot be explained by the few and mild genotype–phenotype correlations identified between *FBN1* mutations and MFS features [3,4,5,6,7,8,9,10,11].

Our team pioneered the hypothesis that MFS severity could be related to the variable level of fibrillin-1 synthesized from the wild-type (WT) allele. To investigate this hypothesis, we firstly established *FBN1* expression in skin fibroblasts from controls as reference values and then in MFS patients [12]. Results in controls showed a 3.9-fold variation in *FBN1* messenger RNA (mRNA) synthesis level. A similar 4.4-fold variation was found in the MFS population, while the mean level of *FBN1* gene expression level was half that of the control population. This study showed also that the *FBN1* gene expression level was a good surrogate endpoint for severity of some MFS clinical features. Indeed, lower *FBN1* gene expression levels were found to be significantly associated with ectopia lentis.

Alternative splicing is an essential biological process, and differential expression of alternative transcripts can be involved in specific biological or pathological roles. A published study on alternative splicing of the *FBN1* gene showed that one *FBN1* isoform’s (no longer referenced in the Ensembl database) expression represented a significant proportion of total *FBN1* gene expression, and that this proportion varied in a tissue- and development-specific manner [13]. Therefore, alternative splicing of the *FBN1* gene could be a potential mechanism that would explain part of the clinical variability observed in MFS. To investigate this hypothesis, the expression profiles of *FBN1* isoforms were investigated firstly in skin fibroblasts from control subjects to establish reference patterns. Expressions of the validated isoforms were then investigated in MFS patients, and possible correlations with clinical features were investigated.

The results of the study showed that, in skin and adventitial fibroblasts, only three isoforms were found: *FBN1_001*, *FBN1_004*, and *FBN1_009*. The main isoform was *FBN1_001* and it was significantly reduced in skin and adventitial fibroblasts from thoracic aortic aneurysm (TAA) patients, as well as in MFS patients. The expressions of *FBN1_004* and *FBN1_009* isoforms were similar between controls and MFS patients, and their expression levels were associated with the presence of ectopia lentis in MFS patients. The alternative splicing mechanism, giving rise to these isoforms, remains unclear; however, these results suggest a role of this mechanism in MFS clinical variability.

## 2. Materials and Methods

### 2.1. Patient and Control Samples

MFS patients were recruited in the “Centre National Maladies Rares—Syndrome de Marfan et apparentés”, the French National Reference Centre located at Bichat Hospital (Paris, France). An in-depth clinical investigation was performed, as well as imaging and slit-lamp ocular examination, to establish a systemic score and perform diagnosis of Marfan syndrome according to the revised Ghent nosology, as already reported in Reference [11]. In brief, the seven different organ systems (skin and integument, skeleton, eye, cardiovascular, neurology, and lung) were carefully examined. For the purpose of this study, adventitial fibroblasts were extracted from five patients with TAA who underwent aortic surgery (two *TGFBR2* mutation carriers, one *FBN1* mutation carrier, and two with aortic aneurysm of undetermined origin). For these patients, skin fibroblasts were also available and were used to investigate the relevance of *FBN1* isoform expression in skin fibroblasts with respect to expression in adventitial fibroblasts. Skin fibroblasts from 42 MFS patients carrying a premature termination codon (PTC) *FBN1* gene mutation were selected as a representative subset of the patient population used in Aubart et al. (2015). To develop and validate the various tests, skin fibroblasts from 15 control subjects were used. All patients gave their written informed consent for participation in this study in agreement with the requirements of French regulations (accepted by the “Comité de Protection des Personnes CPP Ile de France XI”, 78105 St Germain en Laye, with the registration number #11008).

### 2.2. Cell Culture and RNA Purification

Skin fibroblasts were cultivated in Dulbecco’s modified Eagle medium (DMEM; Thermo Scientific, Villebon sur Yvette, France) supplemented with 4.5 g/L glucose, 15% fetal bovine serum (FBS; PAA Laboratory, Villacoublay, France), and antibiotics (50 U/mL penicillin, streptomycin, and amphotericin B) (PAA Laboratory, Villacoublay, France), as previously described [12].

From fibroblast culture, total RNAs were extracted with the RNeasy kit^®^ (Qiagen S.A., Courtaboeuf, France) according to the manufacturer’s instructions, as previously described [14].

### 2.3. Real-Time Quantitative PCR (RT-qPCR)

Reverse transcription was performed using the miScript II RT kit (Qiagen S.A.), according to the manufacturer’s recommendations. The final concentration of complementary DNA (cDNA) was 100 ng/µL for each sample. Real-time quantitative PCR (RT-qPCR) was performed as previously described [14]. Specific primers (Table 1) were used to quantify the gene expression of *FBN1* transcripts, *SDHA*, and *GAPDH*. Total *FBN1* gene expression was calculated by summing the expressions of the three isoforms detected by RT-qPCR.

### 2.4. Statistical Analysis

Statistical analysis was performed using GraphPad Prism version 7.0 software and Jump 7.0.1 software (SAS Institute Inc., Cary, NC, USA). The statistical difference between two groups was tested using an unpaired Student’s *t*-test, while that between three groups was tested using one-way ANOVA or a Kruskal–Wallis test. The coefficient of determination (*R*^2^) was calculated by linear regression, and the statistical significance was determined with Pearson correlation. The significance was set at a *p*-value < 0.05 for all tests.

## 3. Results

### 3.1. Expression of FBN1 Isoforms in Skin Fibroblasts from Controls

**Figure 1:**
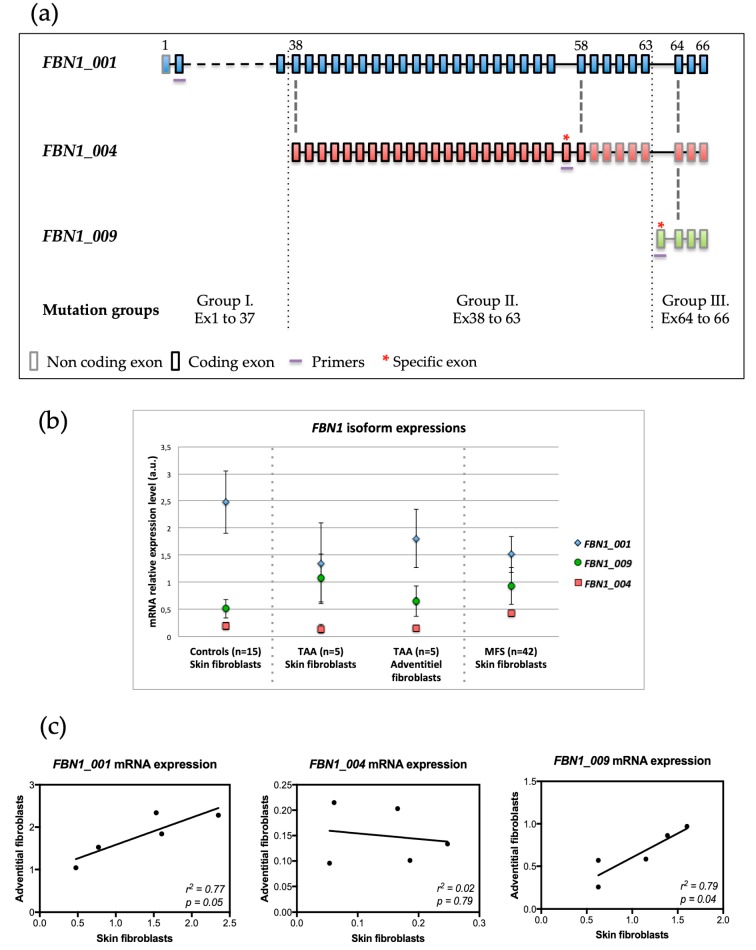
Description of expressed *FBN1* isoforms. (**a**) In silico analysis of *FBN1* isoforms expressed in skin fibroblasts from controls and Marfan syndrome (MFS) patients. Colored boxes represent exons, and coding (boxes with black borders) and non-coding (boxes with gray borders) regions. Exons are numbered in accordance with the referenced sequence (*FBN1_001* isoform) above the boxes. Bold red asterisks indicate exons specific to each isoform. The purple lines indicate the localization of each set of primers used to quantify the expression of each isoform. MFS patients were divided into three groups depending on where the mutation was located: group I (mutations in exons 1 to 37, included), group II (exons 38 to 63, included), and group III (exons 64 to 66, included). (**b**) *FBN1* isoform expressions in skin and adventitial fibroblasts. Quantification using real-time quantitative PCR (RT-qPCR) was performed in skin fibroblasts from 15 controls and 42 MFS patients and in adventitial fibroblasts from five patients with thoracic aortic aneurysm (TAA). (**c**) Correlation of expression between skin and adventitial fibroblasts. Comparison of *FBN1* messenger RNA (mRNA) level for five TAA patients.

In silico analysis of *FBN1* isoforms showed the existence of reports for eight isoforms listed in the Ensembl database [15]. However, none of these isoforms are reported in the literature. In the Genotype-Tissue Expression (GTEx) consortium database [16], the expressions of three of the eight isoforms are reported in transformed fibroblasts and in the aorta: *FBN1_001* (ENST00000316623.5, NM_000138), *FBN1_004* (ENST00000559133.1), and *FBN1_009* (ENST00000561429.1) (Figure 1a). The existence of all eight isoforms in control skin fibroblasts was investigated using RT-qPCR and specific primer pairs. Only isoforms *FBN1_001*, *FBN1_004*, and *FBN1_009* were found. The *FBN1_001* isoform was by far the major isoform found. Furthermore, in our model, *FBN1_009* expression was higher than that of *FBN1_004* (Figure 1b), unlike what is observed in the GTEx database.

### 3.2. Well-Correlated Expression of FBN1 between Skin and Aortic Adventitial Fibroblasts

To investigate isoform expression in adventitial fibroblasts and to compare expression levels between adventitial and skin fibroblasts, we used samples from five TAA patients for whom both cell types were available. The three skin isoforms were also found in adventitial fibroblasts in agreement with GTEx data. Furthermore, skin and aortic expression levels were comparable for all three isoforms with isoform *FBN1_001* as the major isoform (Figure 1b). Finally, a comparison of *FBN1* isoform expressions in adventitial and skin fibroblast pairs showed a positive correlation for *FBN1_001* (*R*^2^ = 0.77; *p* = 0.049) and for *FBN1_009* (*R*^2^ = 0.8; *p* = 0.041). No correlation was found for *FBN1_004* (*R*^2^ = 0.02; *p* = 0.79) (Figure 1c).

### 3.3. Expression of FBN1 Isoforms in Skin Fibroblasts from MFS Patients

**Figure 2:**
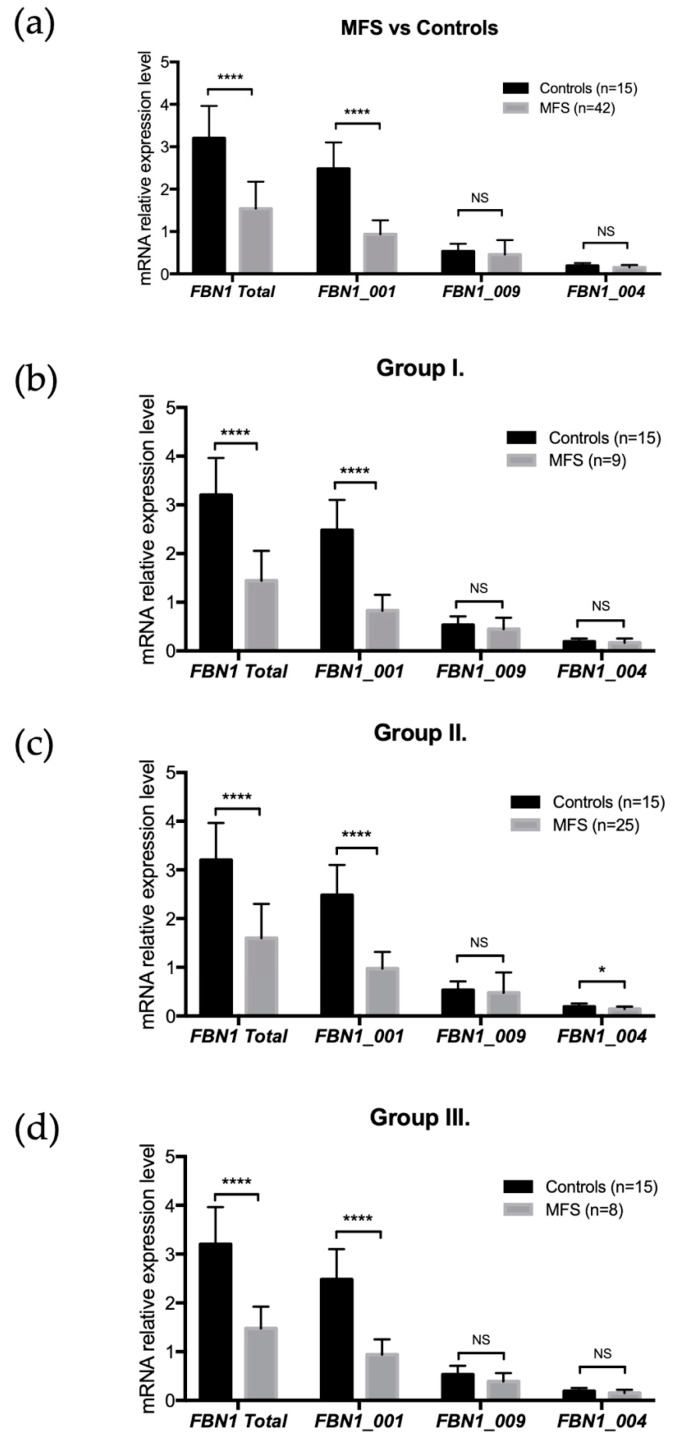
*FBN1* isoform expressions and *FBN1* mutation locations. (**a**) *FBN1* isoform expression levels in controls and MFS patients. A significant decrease in expression was found for MFS patients regarding *FBN1* total expression (*p* < 0.0001) and *FBN1_001* expression (*p* < 0.0001). No significant difference was found for the other two isoforms (*FBN1_004* and *FBN1_009*). (**b**) Isoform expression levels in group I. A significant decrease in expression was found for *FBN1* total expression (*p* < 0.0001) and *FBN1_001* expression (*p* < 0.0001). No difference was observed for *FBN1_004* and *FBN1_009*. (**c**) Isoform expression levels in group II. A significant decrease in expression was found for *FBN1* total expression (*p* < 0.0001), *FBN1_001* expression (*p* < 0.0001), and *FBN1_004* expression (*p* = 0.015). No difference was observed for *FBN1_009*. (**d**) Isoform expression levels in group III. A significant decrease in expression was found for *FBN1* total expression (*p* < 0.0001) and *FBN1_001* expression (*p* < 0.0001). No difference was observed for *FBN1_004* and *FBN1_009*. NS: *p* > 0.05; *: *p* < 0.05; ****: *p* < 0.0001.

In MFS skin fibroblasts, as expected, the total level of *FBN1* mRNA was significantly lower than in control fibroblasts (*p* < 0.0001). The relative expression of isoform *FBN1_001* was significantly reduced (*p* < 0.0001) compared to control (Figure 1b). No significant difference compared to controls was observed for the relative expressions of *FBN1_004* (*p* = 0.064) and *FBN1_009* (*p* = 0.27) (Figure 2a).

### 3.4. FBN1 Isoform Expressions and FBN1 Mutation Locations

The possible impact on *FBN1* isoform expression of the location of the mutation in the *FBN1* gene was tested. MFS patients were divided into three groups: group I (mutations between exons 1 and 37, included), group II (exons 38 to 63, included), and group III (exons 64 to 66, included) (Figure 1a). For all groups compared to controls, a significant decrease in total *FBN1* expression and isoform *FBN1_001* expression was observed (*p* < 0.0001) (Figure 2a–d). *FBN1_004* expression was reduced in group II (*p* = 0.03), while no difference in expression was observed in group III (Figure 2c). No difference in expression was observed for isoform *FBN1_009* in all three groups (Figure 2a–d).

### 3.5. Correlation between FBN1 Isoform Expressions in Skin Fibroblasts from Controls and MFS Patients

**Figure 3:**
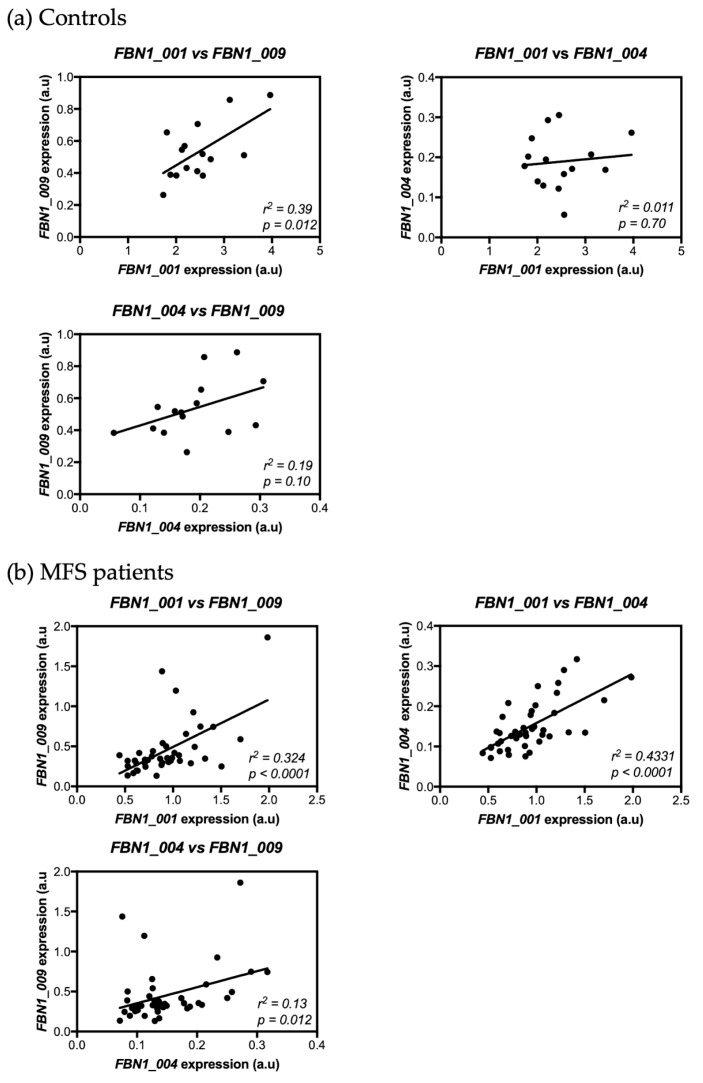
Correlation of expression levels between *FBN1* isoforms. (**a**) Expression levels were assessed in controls. A positive correlation was found between *FBN1_001* and *FBN1_009* expressions. No correlations were found between *FBN1_004* expression and that of the other two isoforms (*FBN1_001* and *FBN1_009*). (**b**) Expression levels were assessed in MFS patients. A positive correlation was found between the three isoforms.

The possible existence of a correlation between *FBN1* isoform expressions was tested both in controls and in MFS patients. In controls, a positive correlation was only observed between the expressions of isoforms *FBN1_001* and *FBN1_009* (*R*^2^ = 0.39, *p* = 0.012) (Figure 3a). Conversely, all isoform expressions were correlated in MFS patients; a positive correlation was found between the expression of isoforms *FBN1_001* and *FBN1_009* (*R*^2^ = 0.32, *p* < 0.0001), between the expression of isoforms *FBN1_001* and *FBN1_004* (*R*^2^ = 0.43, *p* < 0.0001), and between the expression of isoforms *FBN1_004* and *FBN1_009* (*R*^2^ = 0.13, *p* = 0.012) (Figure 3b).

### 3.6. Relationship between FBN1 Isoform Expression and the Clinical Severity of MFS Features

**Figure 4:**
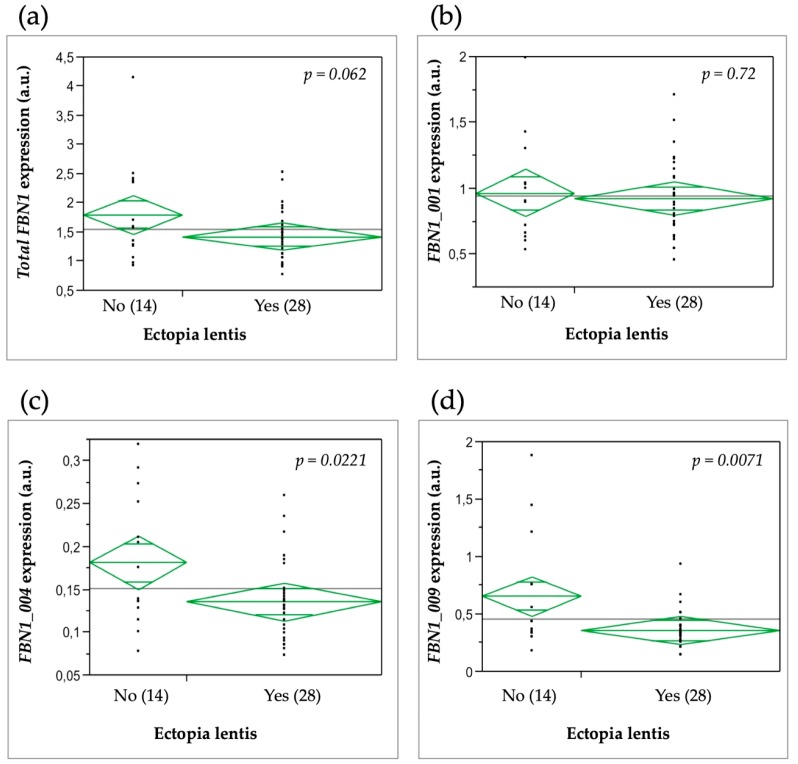
*FBN1* mRNA expression levels in MFS patients with or without ectopia lentis. (**a**) Total *FBN1* expression; (**b**) *FBN1_001* expression; (**c**) *FBN1_004* expression; (**d**) *FBN1_009* expression. Diamond plots display the means of each group. The line across each diamond represents the group mean, and the vertical span of each diamond represents the 95% confidence interval for each group.

Significantly lower levels of *FBN1_004* (*p* = 0.0221) and *FBN1_009* (*p* = 0.0071) expression were found in patients with ectopia lentis as compared to patients without ectopia lentis (Figure 4). No correlation was found between total *FBN1* or *FBN1_001* expression with ectopia lentis. A correlation was also found between *FBN1_009* expression and myopia. Patients with myopia had a higher *FBN1_009* expression compared to patients without myopia (*p* = 0.03). No other relationship was found between *FBN1* isoform expression levels and all other features of the syndrome, notably the cardiovascular, skeletal, and pulmonary systems.

## 4. Discussion

This study investigated, for the first time, the expression profile of *FBN1* isoforms not only in a large number of control subjects, but also in skin and adventitial fibroblast pairs from patients. Previously, Burchett et al. (2011) studied *FBN1* isoform expression profiles in human brain and skeletal muscle, as well as total RNA from human fetal skeletal muscle, brain, liver, aorta, lung, skin, and heart. They detected two splice variants resulting from the inclusion of cryptic exons in intron 54 or 57, but only the latter isoform was further studied. Although this isoform is no longer referenced in the Ensembl database, it could possibly contain the cryptic exon found in *FBN1_004*. However, Burchett et al. (2011) did not identify the 5′ truncation of exons 1 to 37 that leads to isoform *FBN1_004*. The results of our study show that, in skin and adventitial fibroblasts, only three of the eight isoforms referenced in databases exist: *FBN1_001*, *FBN1_004*, and *FBN1_009*. The main isoform is *FBN1_001* and corresponds to the reference transcript. The other two isoforms are shorter. The *FBN1_004* isoform comprises 30 exons (with a specific exon 21), which match the last 29 exons of isoform *FBN1_001*. As per the Ensembl database, no information for its promoter and regulation regions, as well as its start codon, is available, since the 5’ untranslated region (UTR) sequence of the isoform is incomplete. Therefore, the precise reading frame of *FBN1_004* isoform is unknown. Isoform *FBN1_009* comprises four exons with a specific exon 1, and the last three exons of the *FBN1* gene. It is referenced in Ensembl as a processed and non-coding transcript. Its function remains unknown. In our control cell models (adventitial and skin fibroblasts), *FBN1_009* expression was higher than that of *FBN1_004*, contrary to what is reported in the GTEx database. Furthermore, we observed a significant correlation only between the expressions of the two isoforms *FBN1_001* and *FBN1_009*. A single promoter region is described for the *FBN1* gene and its isoforms. This region contains three distinct exons (exons A, B, and C) that are alternatively spliced with the first coding exon of the gene [17]. The three exons are embedded in an approximately 1.8-kb cytosine–phosphate–guanine (CpG) island that contains multiple putative specificity protein 1 (Sp-1) binding sites [18,19]. The promoter that includes exon A appears to be the most commonly used [18,19,20,21] and this may partly explain the positive expression correlation between the two isoforms *FBN1_001* and *FBN1_009.* In the same way, the lack of expression correlation between *FBN1_004* and the other isoforms could result from an alternative splicing mechanism via another promoter exon (B or C).

As previously described [12], total *FBN1* expression was significantly reduced in MFS patients as expected in PTC-mutation carriers. This decrease was only due to a significant decrease in *FBN1_001* expression, while no significant difference was observed for *FBN1_004* and *FBN1_009* expressions. The effect on *FBN1_001* expression was expected since it is the main isoform. Conversely, since these results combine the overall effect of mutations distributed throughout the gene, it is possible that mutations affecting specific exons lead to different isoform expression patterns, notably for isoforms *FBN1_004* and *FBN1_009*. To test this hypothesis, MFS patients were divided into three groups based on the location of the mutation: mutations in the 3′ region common to all three isoforms, mutations between exons 38 and 63 (region common to isoforms *FBN1_001* and *FBN1_004*), and mutations in the 5′ region from exon 1 to 37 (isoform *FBN1_001* exon-specific region). Expression of this last isoform was significantly reduced in all mutation groups. Furthermore, *FBN1_004* expression levels were lower in MFS patients carrying a mutation between exons 38 and 63 (group II) compared to the two other groups of patients, while *FBN1_009* expression showed no difference between mutation groups. These two results could be explained by the presence or absence of nonsense-mediated mRNA decay (NMD) depending on the isoform. NMD is the main biological process for RNA degradation that targets only actively translated mRNAs that carry a PTC [22]. Therefore, this process will not affect isoform *FBN1_009* since it is not translated. However, it should affect isoforms *FBN1_001* and *FBN1_004*. Therefore, NMD could explain the decrease observed in the expression of both these isoforms in MFS patients. Interestingly, the expression of these isoforms is correlated in patients. This could reflect the existence of a specific mechanism that is activated to compensate for the impact of the mutation in the gene.

As previously shown, *FBN1* expression level is a good surrogate endpoint for clinical severity, as lower *FBN1* expression levels are associated with ectopia lentis and pectus excavatum [12]. In our study, we found a trend toward a correlation between total *FBN1* expression and the presence of ectopia lentis. This could be explained by different sample sizes, since we could only test a subset of the original sample in this study and we have, therefore, a lower statistical power. Furthermore, the original study design did not take into account the existence of the three isoforms. Indeed, in Aubart et al. (2015), *FBN1* expression was quantified as a mean of the whole *FBN1* expression evaluated with two sets of primers located in exons 2 and 47. Therefore, correlation analyses were retrospectively performed, firstly, only using expression data obtained with exon 2 primers and, secondly, only using the data from exon 47 primers. This second analysis revealed a correlation with ectopia lentis in agreement with the original results, while no correlation was found with data from exon 2 primers. Therefore, our present results would indicate that the correlation observed with ectopia lentis is supported by isoform *FBN1_004.* Furthermore, a significant association was found in patients between the expressions of *FBN1_004* and *FBN1_009*. This suggests that variations in the expression of both these isoforms have an impact on the pathogenesis of ectopia lentis.

## 5. Conclusions

In conclusion, in our study, we showed, for the first time, that *FBN1* alternative splicing could be a mechanism underlying MFS clinical variability. Further data must now be accrued to determine the sequences involved in the overall regulation of the expression of the *FBN1* gene and its three alternative transcripts.

## Figures and Tables

**Table 1:** Primer sequences for gene expression quantification.

Name	Forward	Reverse
***FBN1_001***	AGTCGGGCCAAGAGAAGAGGCG	TCCATCCAGGGCAACAGTAAGCAT
***FBN1_004***	TTTTACTGCTGTCTCCAGCTTTCC	ACAGCAGCATTCCGATTTGGTG
***FBN1_009***	AAACTCATGGTTTTCCCCCTTCT	TGATGTCTTGGCATCCTCCAC
***GAPDH***	GTCGCCAGCCGAGCCACATC	CCAGGCGCCCAATACGACCA
***SDHA***	AAGGGCTCCGACTGGCTGGGG	TTTCTAGCTCGACCACGGCGGC
